# Real-world effectiveness and safety of hyperthermic intraperitoneal chemotherapy and intraperitoneal chemotherapy in ovarian cancer

**DOI:** 10.1093/oncolo/oyaf424

**Published:** 2025-12-19

**Authors:** Siyi Zhang, Qian Qie, Yuan Zhang, Ye Liang, Siyu Yang, Yiran Wang, Yuqi Wang, Yuanyuan Zhou, Aparna Singh, Yaling Zhao, Qiling Li

**Affiliations:** Department of Obstetrics and Gynecology, The First Affiliated Hospital of Xi’an Jiaotong University, Xi’an 710061, People’s Republic of China; Department of Obstetrics and Gynecology, The First Affiliated Hospital of Xi’an Jiaotong University, Xi’an 710061, People’s Republic of China; Department of Obstetrics and Gynecology, The First Affiliated Hospital of Xi’an Jiaotong University, Xi’an 710061, People’s Republic of China; Department of Obstetrics and Gynecology, The First Affiliated Hospital of Xi’an Jiaotong University, Xi’an 710061, People’s Republic of China; Department of Obstetrics and Gynecology, The First Affiliated Hospital of Xi’an Jiaotong University, Xi’an 710061, People’s Republic of China; Department of Obstetrics and Gynecology, The First Affiliated Hospital of Xi’an Jiaotong University, Xi’an 710061, People’s Republic of China; Department of Obstetrics and Gynecology, The First Affiliated Hospital of Xi’an Jiaotong University, Xi’an 710061, People’s Republic of China; Department of Pathology, The First Affiliated Hospital of Xi’an Jiaotong University, Xi’an 710061, People’s Republic of China; Department of Obstetrics and Gynecology, The First Affiliated Hospital of Xi’an Jiaotong University, Xi’an 710061, People’s Republic of China; Department of Epidemiology and Biostatistics, School of Public Health, Xi’an Jiaotong University Health Science Center, Xi’an 710061, People’s Republic of China; Department of Obstetrics and Gynecology, The First Affiliated Hospital of Xi’an Jiaotong University, Xi’an 710061, People’s Republic of China

**Keywords:** ovarian cancer, hyperthermic intraperitoneal chemotherapy, intraperitoneal chemotherapy, real-world study

## Abstract

**Background:**

Both hyperthermic intraperitoneal chemotherapy (HIPEC) and conventional intraperitoneal chemotherapy (IP) have shown survival benefits in ovarian cancer (OC), but direct comparisons between the two perfusion modalities are lacking. This study aimed to compare effectiveness and safety between HIPEC and conventional IP in OC.

**Methods:**

This retrospective real-world study analyzed 606 patients with stages II-IV OC who received HIPEC or IP following cytoreductive surgery between 2013 and 2024. The primary endpoint was progression-free survival. Overall survival and adverse events were secondary endpoints. The study used inverse probability of treatment propensity-score weighting. We also conducted sensitivity analyses to evaluate result robustness and subgroup analyses to explore potential effect modification.

**Results:**

After a median follow-up of 26 months, disease progression occurred in 40.6% of patients in the HIPEC group and 55.0% in the IP group (hazard ratio [HR] 0.79; *P* = .103). Mortality rates were 13.2% and 22.5%, respectively (HR 0.83; *P* = .434), showing no significant differences in progression and survival between the two groups. Exploratory subgroup analyses suggested a trend toward improved progression-free outcomes with HIPEC, particularly among patients with BRCA wild-type or BRCA1-mutated tumors and early postoperative perfusion. Hypoalbuminemia was the most common event in both groups (HIPEC 27.2%; IP 15.6%). HIPEC group had more abdominal distension and wound dehiscence, whereas IP patients experienced nausea and rash more frequently.

**Conclusions:**

HIPEC did not significantly improve survival over conventional IP in the overall population, but showed greater benefit in specific subgroups, underscoring the importance of individualized intraperitoneal chemotherapy strategies in OC.

Implications for Practice This study presents the first direct comparison between HIPEC and conventional IP in ovarian cancer. While no overall survival difference was observed, exploratory analyses suggested greater benefit from HIPEC in selected subgroups, particularly those with BRCA wild-type or BRCA1-mutated tumors and early postoperative perfusion. These findings provide crucial evidence for individualized intraperitoneal chemotherapy strategies, offering valuable insights for future trial design.

## Introduction

Ovarian cancer (OC) is the eighth most prevalent malignancy among women and the second leading cause of gynecologic cancer death (after cancer of the cervix uteri) worldwide. Approximately 324 398 new cases (3.4% of all cancer cases) and 206 839 deaths (4.8% of total cancer deaths) emerged in 2022.^1–3^ In China, the incidence and mortality rates of OC in 2022 arrived at 61.1/100 000 and 32.6/100 000, respectively.^4^^,^^5^ It is estimated that roughly 75% of patients are diagnosed at advanced stages, when the tumor has already disseminated extensively into peritoneal cavity.^6^

Given OC’s propensity for peritoneal spread, intraperitoneal chemotherapy (IP) has been considered an ideal option.^7^^,^^8^ By delivering drugs directly into peritoneal cavity, intraperitoneal administration achieves higher drug concentration at tumor sites while lower systemic side effects than intravenous chemotherapy concurrently. Following the publication of the pivotal trial (GOG 172),^9^ the National Cancer Institute released a clinical alert in 2008 recommending IP as a treatment option for patients with stage III OC who have undergone optimally debulking surgeries.^10^^,^^11^ IP administration enhances drug delivery and eliminates residual microscopic disease along the peritoneal surface.^9^^,^^12^^,^^13^ However, the penetration depth of IP is restricted to a range of 1-2 mm, implying that its benefit is confined to patients with microscopic or extremely small volume residual disease following debulking surgeries.^14^

Hyperthermic intraperitoneal chemotherapy (HIPEC) introduces instillation of heated chemotherapy (typically at 41-43 °C for 30-120 min) into the abdominal cavity,^15^ and utilizes the effect of hyperthermia to improve drug penetration at the peritoneal surface (increasing the depth penetration to about 3 to 5 mm). Beyond the hyperthermic effect, HIPEC also generates mechanical stimulation through fluid shear stress. The perfusate circulates continuously within the peritoneal cavity at a controlled flow rate, using larger volumes and producing higher instantaneous drug exposure.^16^ Conventional IP uses smaller volumes that remain static in the cavity for a longer dwell duration. Pharmacokinetically, HIPEC delivers a single high-dose perfusion with limited systemic absorption,^15^ whereas IP involves multiple perfusions with lower per-cycle dose intensity but greater cumulative exposure over time.^17^ HIPEC also promotes the formation of cisplatin-DNA strand adducts,^18^ impairs the intrinsic homologous recombination DNA repair pathway,^15^ and improves tumor perfusion to counteract hypoxic tumor microenvironment that is often linked to chemotherapy resistance.^19–24^ Additionally, hyperthermia induces apoptosis, activates heat-shock proteins that act as natural killer cell receptors, suppresses angiogenesis, and promotes protein denaturation.^18^^,^^25^^,^^26^ Multiple studies indicate that the addition of HIPEC treatment is consistently associated with survival advantages when contrasted with cytoreductive surgery (CRS) only.^15^^,^^27^^,^^28^ Based on available evidence, the 2019 NCCN Guidelines recommend for the first time the use of HIPEC during interval CRS for stage III OC patients.^29^

Both HIPEC and IP have demonstrated survival benefits in OC. However, up to now, there is no study comparing the two methods,^15^^,^^27^^,^^28^^,^^30^ which is critical to identifying their suitable crowds and appropriate working conditions. Whether the effect of HIPEC is due to the effect of hyperthermia, the intraperitoneal administration of chemotherapy, or some other factors, remains unanswered. To address this gap, we carried out a retrospective real-world study comparing the effectiveness and safety of postoperative HIPEC and IP in the treatment of OC.

## Methods

### Study design

This single-center retrospective real-world study was conducted at the First Affiliated Hospital of Xi’an Jiaotong University. Between August 2013 and March 2024, 606 patients were included for analysis. The date of the last follow-up was January 25, 2025, and the data were locked on March 1, 2025. The present study was reviewed and approved by our Institutional Review Board (XJTU1AF2024LSYY-469) in accordance with the Declaration of Helsinki and the International Conference on Harmonisation Good Clinical Practice guidelines.

The extent of peritoneal carcinomatosis was quantified using the Peritoneal Cancer Index (PCI) according to Sugarbaker’s classification.^31^ PCI assessment was performed intraoperatively during open laparotomy, after complete adhesiolysis and full visualization of all visceral and parietal peritoneal surfaces. The abdominal cavity is divided into 13 regions, and each region is scored by lesion size, ranging from 0 to 3 according to the largest visible tumor nodule within that region (Table S1). The PCI is calculated as the sum of the 13 regional lesion size scores, reflecting the overall extent of peritoneal involvement. To achieve optimal tumor reduction, any lesion or adhesion suspected of potential metastasis was selectively resected. Hospital stay was defined as the days between the date of surgery and the date of discharge.

### Treatments

Administration of HIPEC or IP was done 1-9 days postoperatively.

HIPEC: The BR-TRG-II hyperthermic intraperitoneal perfusion system (Baorui Medical Technology, China) was used under a closed-abdomen circulation mode. The perfusate was 0.9% sodium chloride 4000 mL at a constant temperature of 43 °C (109.4 °F). The procedure took 60 min with a flow rate of 400 mL/min. Cisplatin (70 mg/m^2^ body surface area) or lobaplatin (50 mg/m^2^) was used as the chemotherapeutic agent. A total of 3-5 postoperative perfusions were performed, of which 1-2 sessions contained chemotherapy, while the remaining sessions used saline only for thermal lavage. After each session, drainage tubes were opened to evacuate the perfusate, and the abdominal catheters were removed following the final session.

IP: The IP regimen used the same chemotherapeutic drugs and doses as the HIPEC protocol. The perfusate contained 5 mL of lidocaine and 5 mg of dexamethasone dissolved in 500 mL of 0.9% sodium chloride. The solution was instilled into the peritoneal cavity and retained for 6 hours before drainage. IP was performed 1-2 sessions but under normothermic, static dwell conditions without continuous circulation or mechanical perfusion.

For those receiving cisplatin perfusion, patients received adequate hydration and urine output was maintained above 0.5 mL/(kg·h) on the day of treatment to minimize nephrotoxicity. Sodium thiosulfate was not routinely administered during the study period.

### Patients

Potentially eligible patients were identified through the inpatient record system and consented at telephone follow-up.

Inclusion criteria: ① patients with clinically diagnosed stages IIb-IV OC (Table S2). Stage IVb patients included only when extra-peritoneal disease was limited to resectable sites, such as inguinal or para-aortic lymph nodes or pleural effusion; ② patients receiving optimal CRS with residual disease at R0 or R1 level (Table S3); ③ patients undergoing HIPEC or IP following CRS. Patients who met al. three of the above were included in the study.

Exclusion criteria: ① patients pathologically diagnosed as other metastatic cancers; ② patients receiving incomplete CRS, such as laparoscopic biopsy, open surgery without resectable lesions or with residual disease at R2 level; ③ patients with unresectable distant metastases (lung parenchymal lesions, brain, bone, or distant lymph nodes); ④ patients who refused to participate in this study during telephone follow-up.

### End points

The primary end point was progression-free survival (PFS), which was defined as the time between the initiation of HIPEC or IP and the date of disease progression or death from any cause. Disease progression was defined by a clinical evidence that was based on radiologic findings of tumor growth according to Response Evaluation Criteria in Solid Tumors, version 1.1,^32^ or biochemical assessment of the Gynecologic Cancer Inter Group, whichever occurred first.^33^ Patients in both the HIPEC and IP groups underwent follow-up every 3 months during the first 2 years and every 6 months thereafter, including physical examination, ultrasound and serum CA-125 measurement at each scheduled visit. Imaging (CT or MRI) was assessed annually according to institutional practice. Secondary end points involved adverse event (AE) profile and overall survival (OS), which was calculated from the initiation of HIPEC or IP to the date of death from any cause. AEs were evaluated from the date of HIPEC or IP and thereafter 21 days according to the National Cancer Institute Common Terminology Criteria for Adverse Events, version 4.0, and complications grade 3 or 4 were considered as severe events. Telephone contact was used to confirm patient participation, complement hospital records and to update survival outcomes at the last follow-up, but did not replace scheduled clinical or radiologic assessments. Outcome adjudication was not formally blinded given the retrospective design.

### Statistical analysis

In the main patient-level analysis, inverse probability of treatment weighting (IPTW) based on propensity scores (PS) was performed to create a weighted cohort in which patients differed by intraperitoneal perfusion methods (HIPEC vs IP) but were balanced across observed baseline characteristics.^34^^,^^35^ PS were estimated using logistic regression. The model regressed treatment assignment on prespecified covariates, including age, tumor classification, disease stage, recurrence status, PCI, interval from surgery to intraperitoneal perfusion treatment, tumor size, surgery duration, perfusion drugs, neoadjuvant chemotherapy regimen and response, which were considered potential confounders of the treatment-outcome relationship. Each patient in the HIPEC group was assigned a weight of 1 ÷ PS, and in the IP group was assigned a weight of 1 ÷ (1 − PS).^36^ To reduce the influence of extreme weights and improve estimate accuracy, we implemented stabilized weights by multiplying the conventional IPTW weights by the marginal probability of receiving the treatment actually received.^37^ Covariate balance between treatment groups after weighting was assessed using absolute standardized mean differences (SMD), with a threshold of ≤0.1 indicating sufficient balance. This approach yields marginal (population-averaged) treatment effect estimates that are less prone to residual confounding compared to conventional multivariable regression and are more analogous to estimates derived from randomized clinical trials.^37^^,^^38^

We compared baseline characteristics between groups in both the unweighted and IPTW cohorts. Continuous variables were analyzed using the Wilcoxon rank-sum test, and categorical variables were compared using the chi-square test in the unweighted cohort. In the weighted cohort, comparisons used IPTW-weighted logistic regression with survey adjustment. Covariate balance after weighting was confirmed by evaluating SMDs. OS and PFS were compared between groups in the weighted cohort. Survival functions were estimated using IPTW-adjusted Kaplan–Meier methods and presented with 95% confidence intervals (CI). Risk tables represented weighted patient counts at risk (not raw integers). Treatment differences were assessed using the IPTW-weighted log-rank tests. Hazard ratios (HR) and 95% CIs of death and disease progression were estimated with IPTW-weighted Cox proportional-hazards models. The restricted mean survival time at 36 months (RMST_36_) was calculated for each treatment group to provide an absolute measure of survival benefit.

Several sensitivity analyses evaluated result robustness. First, to address potential residual confounding from covariates showing imbalance after IPTW (SMD > 0.1), Cox models was adjusted for these variables as additional covariates. Second, multiple imputation via chained equations addressed missing data, followed by repeated IPTW-weighted survival models on the imputed data. Third, post-treatment variable (maintenance therapy, postoperative time to first IV chemotherapy and postoperative chemotherapy courses) were incorporated in doubly robust weighted Cox models to assess the robustness of treatment effects. Fourth, PFS was re-estimated using only radiologically confirmed progression events. In addition, alternative analytic strategies were conducted to verify the consistency of treatment effects, including multivariable Cox regression and a propensity score matching (nearest-neighbor matching without replacement). These results were compared with those of the primary IPTW analysis to evaluate potential mediation effects.

To explore potential effect modification, we conducted subgroup analyses stratified by patient age (<55- vs ≥ 55-year old), stage, histology, primary/recurrent, the presence or absence of neoadjuvant chemotherapy, PCI (<10 vs ≥ 10), interval between surgery and HIPEC/IP, perfusion drugs, perfusion times and BRCA/HRD status. Within each subgroup, IPTW-weighted Cox models estimated HRs (95% CIs) for PFS, summarized using a forest plot. Interaction terms between treatment and subgroup variables were tested in weighted Cox models, with multiplicity addressed by Benjamini–Hochberg correction.

Weighted incidences of AEs were compared using IPTW. Each AE was categorized as either any grade or severe events (grade ≥ 3). Group differences of weighted AE incidences were estimated using survey-adjusted means and performed weighted chi-square tests (Rao–Scott adjusted tests). Results were summarized as weighted proportions with corresponding *P*-values.

## Results

### Patients characteristics

**Figure 1.**
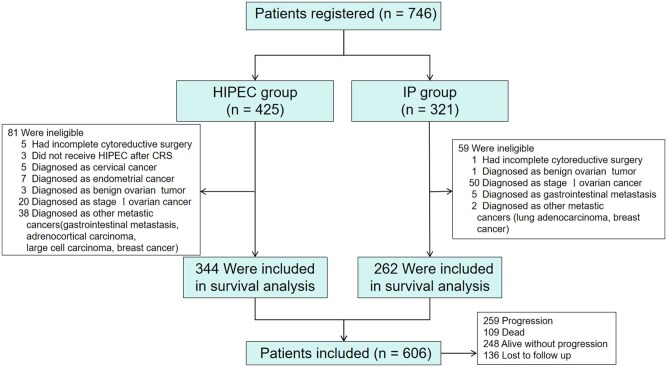
The flow diagram of patient selection. Incomplete cytoreductive surgery was defined as surgery that resulted in the presence of one or more residual lesions measuring more than 10 mm in diameter. HIPEC, hyperthermic intraperitoneal chemotherapy; IP, intraperitoneal chemotherapy.

The flow diagram of patient selection is shown in Figure 1. A total of 606 patients were included in the study cohort, with 344 in the HIPEC group and 262 in the IP group. After IPTW adjustment, the effective sample sizes were 335 for HIPEC and 269 for IP. Baseline demographic and clinical characteristics before and after weighting are summarized in Table 1. Before weighting, the groups showed differences in disease stage and surgical duration. Patients in the HIPEC group had a higher proportion of stage IV disease (26.7% vs 16.4%) and underwent longer operations (median, 190 vs 140 min). Additionally, significantly more HIPEC patients received treatment within 1 day after surgery (64.8% vs 36.6%). Patients in the HIPEC group were more frequently treated with lobaplatin (68.3% vs 36.3%), and underwent a single perfusion cycle compared with the IP group (81.1% vs 68.3%). After IPTW, all baseline characteristics were well balanced between groups (all *P* > .05), indicating effective covariate balancing (Figures S1-S3). Table S4 lists additional baseline characteristics before and after weighting.

**Table 1.** Baseline patient characteristics and treatment information^a^.

	Cohort before inverse probability of treatment weighting			Cohort after inverse probability of treatment weighting		
Variable	HIPEC (*N* = 344)	IP (*N* = 262)	*P* value^b^	HIPEC (*N* = 335)	IP (*N* = 269)	*P* value^c^
**Baseline charateristics**						
**Median age (IQR), yr**	58 (50-64)	56 (48-63)	.304	58 (50-64)	58 (49-63)	.644
**Tumor histoligic type, no. (%)**			.299			.292
**High-grade serous**	293 (85.2)	218 (83.2)		275 (82.0)	226 (84.1)	
**Low-grade serous**	9 (2.6)	13 (5.0)		8 (2.2)	11 (4.1)	
**Endometrioid**	12 (3.5)	8 (3.1)		15 (4.6)	7 (2.7)	
**Mucinous**	8 (2.3)	4 (1.5)		10 (3.0)	4 (1.5)	
**Clear-cell carcinoma**	9 (2.6)	5 (1.9)		13 (3.8)	3 (1.1)	
**Carcinosarcoma**	8 (2.3)	9 (3.4)		10 (2.8)	9 (3.2)	
**Others**	5 (1.5)	5 (1.9)		5 (1.6)	9 (3.3)	
**Primary/Recurrent, no. (%)**			.126			.700
**Primary**	330 (95.9)	243 (92.7)		309 (92.1)	251 (93.4)	
**Recurrent**	14 (4.1)	19 (7.3)		26 (7.9)	18 (6.6)	
**PCI** ^d^ **, no. (%)**			.684			.681
**PCI < 5**	76 (22.1)	68 (26.0)		79 (23.6)	74 (27.5)	
**5 ≤ PCI < 10**	140 (40.7)	102 (38.9)		140 (41.7)	113 (42.2)	
**10 ≤ PCI < 15**	92 (26.7)	69 (26.3)		84 (25.2)	64 (23.6)	
**PCI ≥ 15**	36 (10.5)	23 (8.8)		32 (9.5)	18 (6.6)	
**Stage** ^e^ **, no. (%)**			.006			.995
**II**	27 (7.8)	33 (12.6)		34 (10.1)	29 (10.9)	
**III**	209 (60.8)	167 (63.7)		199 (59.2)	158 (58.8)	
**IV**	92 (26.7)	43 (16.4)		75 (22.5)	64 (23.6)	
**Treatment characteristics**						
**Median duration of surgery (IQR), min**	190 (155-240)	140 (105-180)	<.001	175 (140-215)	170 (120-257)	.879
**Residual disease after surgery, no. (%)**			.127			.079
**R-0, no visible tumor**	335 (97.4)	248 (94.7)		327 (97.6)	250 (93.1)	
**R-1, tumor nodules ≤1 cm**	9 (2.6)	14 (5.3)		8 (2.4)	19 (6.9)	
**Median duration of hospitalization** ^f^ **(IQR), days**	8 (7-11)	8 (6-11)	.054	8 (7-11)	9 (7-11)	.805
**Interval between surgery and start of HIPEC/IP, no. (%)**			<.001			.156
**≤ 1 day**	223 (64.8)	96 (36.6)		180 (53.6)	121 (45.1)	
**> 1 day**	121 (35.2)	166 (63.4)		156 (46.4)	148 (54.9)	
**Perfusion drugs, no. (%)**			<.001			.615
**Cisplatin**	89 (25.9)	155 (59.2)		134 (40.0)	119 (44.4)	
**Lobaplatin**	235 (68.3)	95 (36.3)		185 (55.1)	139 (51.8)	
**Others**	20 (5.8)	12 (4.6)		17 (4.9)	10 (3.8)	
**Perfusion times, no. (%)**			<.001			.845
**1**	279 (81.1)	179 (68.3)		247 (73.5)	200 (74.4)	
**2**	65 (18.9)	83 (31.7)		89 (26.5)	69 (25.6)	

a Due to rounding error, in the weighted cohort counts may not sum to expected totals, and percentages may not be equal to ratios of counts.

b *P* values calculated from chi-square test or Wilcoxon rank-sum test.

c *P* values calculated from inverse probability of treatment-weighted logistic regression models.

d Details on the PCI are provided in Table S1.

e Staging statistics for primary tumors only. Details on the International FIGO staging system are provided in Table S2.

f Duration of hospitalization was calculated from surgery to hospital discharge.

Abbreviations: HIPEC, hyperthermic intraperitoneal chemotherapy; IP, intraperitoneal chemotherapy; IQR, interquartile range; PCI, peritoneal cancer index.

### Efficacy

At the time of data cutoff, the median follow-up duration in the weighted cohort was 26.0 months (IQR 15.1-38.8 months), and it was 4 months longer in the IP group (27.5 months; IQR 16.2-48.7 months) than HIPEC group (23.5 months; IQR 15.1-35.3 months).

**Figure 2.**
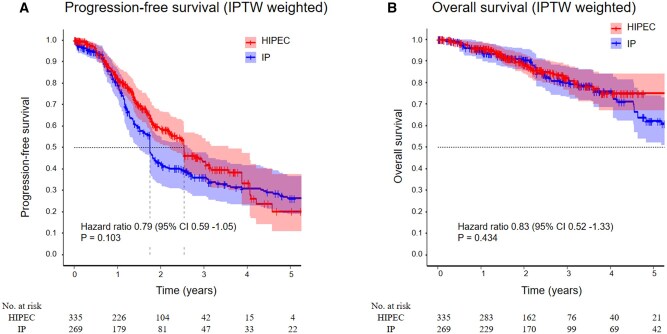
Inverse probability of treatment-weighted Kaplan–Meier in overall population. Kaplan–Meier curves for (A) progression-free survival and (B) overall survival after inverse probability of treatment weighting (IPTW). Shaded bands represent the 95% confidence intervals. Patients who received HIPEC showed a trend toward improved progression-free survival compared with those treated with IP (*P* = .103), though no statistically significant difference was observed. Overall survival was also similar between the two groups (*P* = .434). The at-risk tables below each plot indicate the number of patients at risk at each time point. HIPEC, hyperthermic intraperitoneal chemotherapy; IP, intraperitoneal chemotherapy.

Totally 136 patients (40.6%) in the HIPEC group and 148 patients (55.0%) in the IP group experienced disease progression. HIPEC treatment showed a nonsignificant trend toward reduced progression (HR 0.79, 95% CI 0.59-1.05, *P* = .103; Figure 2A). Median PFS was longer in the HIPEC group (30.5 months, 95% CI 26.5-42.8 months) than in the IP group (21.0 months, 95% CI 18.1-31.5 months). The 3-year PFS rate was 43.4% (95% CI 34.8-54.1) in the HIPEC group compared with 36.1% (95% CI 27.4-47.6) in the IP group. Consistently, the RMST_36_ was longer with HIPEC (25.9 vs 22.8 months). Death occurred in 44 patients (13.2%) in the HIPEC group and 61 patients (22.5%) in the IP group (HR 0.83, 95% CI 0.51-1.33, *P* = .434; Figure 2B). The 3-year OS rates were 82.2% (95% CI 76.6-88.3) for HIPEC and 80.0% (95% CI 73.2-87.4) for IP, and the RMST_36_ was similar between groups (33.1 vs 32.9 months).

**Figure 3.**
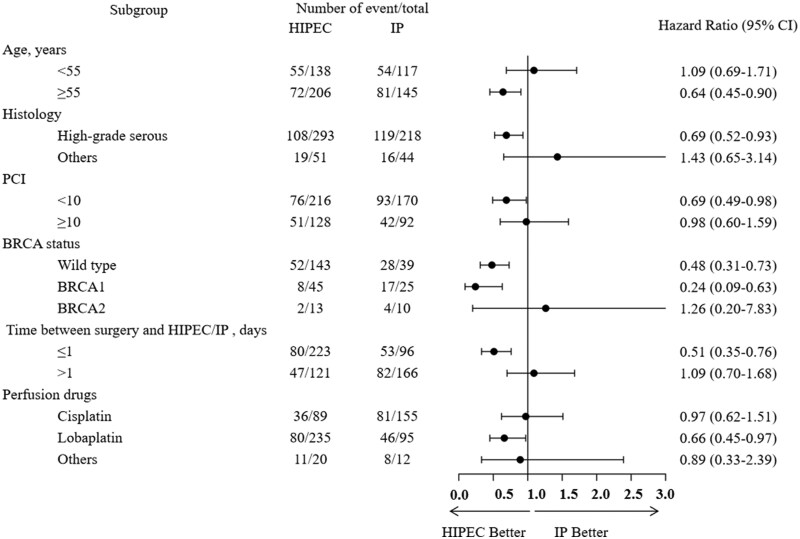
Subgroup analyses of progression-free survival. Exploratory subgroup analyses showing the associations between treatment strategy (HIPEC vs IP) and progression-free survival across various clinicopathological subgroups. Circles represent hazard ratio point estimates comparing HIPEC with IP within each subgroup, and horizontal lines indicate the corresponding 95% confidence intervals. Separate Cox proportional-hazards models weighted by inverse probability of treatment (IPTW) were used for each subgroup. Most subgroups (particularly, perfusion timing ≤ 1 day and BRCA-wild/BRCA1-mutated status) showed HRs below 1.0, indicating a consistent trend toward reduced progression risk with HIPEC. HIPEC, hyperthermic intraperitoneal chemotherapy; IP, intraperitoneal chemotherapy; PCI, peritoneal cancer index.

Exploratory subgroup analyses were performed to evaluate the consistency of treatment effects across different patient populations (Figure 3). Most subgroups showed HRs below 1.0, indicating an overall trend toward reduced progression risk with HIPEC. Numerically greater benefits were observed among patients aged ≥55 years, with high-grade serous histology, lower PCI (<10), BRCA wild-type or BRCA1-mutated tumors, and early postoperative perfusion (≤1 day). No clear treatment differences were observed across other subgroups, including disease stage, recurrence status, neoadjuvant chemotherapy, or perfusion times (Figure S4). Interaction *P* values showed no significant modification of treatment effect by any patient or tumor characteristic. But the consistent trends across several subgroups (particularly, perfusion timing and BRCA status) suggest therapeutic effect heterogeneity between HIPEC and IP in specified populations, which deserves to be validated in larger, prospective trials.

In sensitivity analyses, the findings remained consistent across multiple analytic approaches. After adjusting for unbalanced covariates (age, surgery time, treatment interval and neoadjuvant chemotherapy response) with IPTW, HIPEC was associated with a nonsignificant reduction in progression risk (HR 0.77, 95% CI 0.56-1.05, *P* = .096). Results were similar after accounting for missing data using multiple imputation (HR 0.77, 95% CI 0.58-1.03, *P* = .076). In additional doubly robust sensitivity analyses incorporating post-treatment variables, the findings remained consistent with the primary IPTW analysis (HR 0.84, 95% CI 0.59-1.18, *P* = .306). When restricting progression events to radiologically confirmed RECIST 1.1 progression only, the results were similar and not statistically significant (HR 1.02, 95% CI 0.70-1.49, *P* = .920). Alternative analytic strategies yielded consistent results, including multivariable Cox regression after model selection (HR 0.76, 95% CI 0.57-1.00, *P* = .05; Table S5) and propensity-score matching (HR 0.82, 95% CI 0.60-1.11, *P* = .195; Figures S5 and S6). These sensitivity analyses supported the robustness of the primary findings.

### Safety

Within 21 days after treatment, AEs of any grade occurred in 58.7% of HIPEC patients and 42.4% of IP patients (Table 2). After IPTW adjustment, the HIPEC group had more abdominal distension (37 [11.1%] vs 9 [3.5%]; *P* = .001) and wound dehiscence (7 [2.0%] vs 0; *P* = .011) events than IP. Hypoalbuminemia (91 [27.2%] vs 42 [15.6%]), anemia (54 [16%] vs 26 [9.5%]), upper respiratory infection (28 [8.5%] vs 10 [3.6%]) and blood bilirubin increase (23 [7.0%] vs 10 [3.7%]) also showed higher frequencies in the HIPEC group, though not statistically significant. IP patients experienced nausea (26 [7.9%] vs 41 [15.1%]; *P* = .057) and rash (0 vs 4 [1.6%]; *P* = .056) more frequently. Grade 3 or 4 AEs were relatively rare in both groups. The most common severe event was anemia (HIPEC: 9 [2.7%] vs IP: 14 [5.3%]; *P* = .131). Additional uncommon AEs are listed in Table S6. The median number of postoperative chemotherapy cycles was similar between groups (6 cycles, *P* = .778).

**Table 2.** Common adverse events^a^.

Adverse events	Any grade			Grade 3 or 4		
	No. (%)		*P* value^b^	No. (%)		*P* value^b^
	HIPEC (*N* = 335)	IP (*N* = 269)		HIPEC (*N* = 335)	IP (*N* = 269)	
**Hypoalbuminemia**	91 (27.2)	42 (15.6)	.115	1 (0.2)	1 (0.2)	.940
**Anemia**	54 (16.0)	26 (9.5)	.052	9 (2.7)	14 (5.3)	.131
**Nausea**	26 (7.9)	41 (15.1)	.057	0	0	
**Abdominal distension**	37 (11.1)	9 (3.5)	.001	2 (0.5)	0	.216
**Aspartate aminotransferase increased**	21 (6.2)	28 (10.3)	.190	1 (0.4)	0	.201
**Alanine aminotransferase increased**	19 (5.7)	26 (9.7)	.188	0	0	
**Upper respiratory infection**	28 (8.5)	10 (3.6)	.090	1 (0.3)	0	.372
**Abdominal pain**	19 (5.6)	19 (6.9)	.615	1 (0.4)	0	.212
**Blood bilirubin increased**	23 (7.0)	10 (3.7)	.186	0	3 (1.0)	.184
**Vomiting**	16 (4.7)	10 (3.8)	.655	1 (0.2)	0	.373
**Hypocalcemia**	19 (5.7)	11 (4.1)	.553	6 (1.8)	1 (0.5)	.108
**Fever**	19 (5.6)	13 (4.8)	.758	0	0	
**Hypokalemia**	12 (3.6)	10 (3.7)	.970	1 (0.3)	0	.373
**Cough**	12 (3.7)	5 (1.9)	.333	0	0	
**Creatinine increased**	10 (3.0)	6 (2.4)	.677	0	0	
**Wound dehiscence**	7 (2.0)	0	.011	6 (1.9)	0	.016

a Data are given for adverse events that occurred ≥5% in any grade, ≥1%in grade 3/4, from the initiation of intraperitoneal chemotherapy and thereafter 21 days, and are listed in descending order of frequency in the all patients. The adverse events were graded according to the National Cancer Institute Common Terminology Criteria for Adverse Events, version 4.0 Additional adverse events are provided in Table S6 in the Supplement.

b Group differences of weighted event rates estimated using survey-adjusted means and performed weighted chi-square tests.

Abbreviations: HIPEC, hyperthermic intraperitoneal chemotherapy; IP, intraperitoneal chemotherapy; CI, confidence interval.

## Discussion

Our real-world study of 606 patients suggests that HIPEC was not associated with a statistically significant improvement in PFS or OS compared to conventional IP in the overall population. This lack of significance remained consistent across multiple analytic approaches, including IPTW adjustment, multivariable Cox regression, propensity score matching, and doubly robust weighted Cox models. However, exploratory subgroup analyses indicated potential efficacy heterogeneity, with HIPEC showing a greater benefit trend than IP among patients with early initiation of intraperitoneal perfusion (within 1 day after surgery) and specified molecular characteristics (BRCA wild-type or BRCA1-mutated tumors). These findings suggest potential differential treatment effects and underscore the importance of individualized therapeutic strategies in OC.

To our knowledge, this is the first study directly comparing the efficacy and safety of HIPEC with IP in patients with OC. HIPEC gained widespread adoption after the publication of the 2018 sentinel clinical trial (OVHIPEC-1), which demonstrated significant improvements in PFS (by 3.5 months) and OS (by 11.5 months) for patients undergoing interval CRS with HIPEC.^15^ Prior to that, IP was the predominant mode of intraperitoneal therapy. Both HIPEC and IP have shown advantages over CRS alone,^9^^,^^10^^,^^15^^,^^27^^,^^39^ but few studies have directly compared these two perfusion modalities, leaving a critical gap in comparative data. A network meta-analysis reported superior survival for HIPEC over intravenous chemotherapy, but no significant differences between HIPEC and IP.^40^ The ongoing HyNOVA trial aims to compare HIPEC with normothermic IP, but results have not yet been reported.^41^

Theoretically HIPEC would have a better effectiveness because it enhances the penetration of chemotherapeutic agents^42^ and induces tumor cytotoxicity.^22^ However, our findings challenge the prevailing assumption of HIPEC’s superiority. Several factors may explain the lack of significant survival benefit. First, the two intraperitoneal chemotherapy approaches in this study differed in multiple procedural aspects beyond temperature, including perfusion technique, session number, perfusate volume, and chemotherapeutic regimen. These variations between the two intraperitoneal protocols may have attenuated the theoretical advantage of hyperthermia and contributed to the overlapping efficacy between HIPEC and IP. Second, with optimal CRS, conventional IP may achieve adequate disease control, diminishing the relative benefit of HIPEC. Third, the therapeutic effects demonstrate consistent heterogeneity across subgroup populations, with variations attributable to tumor molecular characteristics, initiation timing of treatment, and histopathological classifications.

Our exploratory subgroup analysis suggests that therapeutic effect difference between HIPEC and IP is possibly influenced by perfusion timing and tumor molecular characteristics. Patients who received perfusion within 1 day after surgery tended to have more favorable outcomes. This finding aligns with prior evidence that immediate postoperative perfusion maximizes drug exposure to the peritoneal surface and enhances cytotoxic penetration under hyperthermic conditions. Residual tumor cells are more sensitive to hyperthermia at the early postoperative stage, when surgical trauma induces transient local hypoxia and places the cells into a quiescent (G0) phase.^43^^,^^44^ In addition, HIPEC appeared more beneficial than IP in BRCA wild-type and BRCA1-mutated tumors, but not clearly in BRCA2-mutated disease. Loss of BRCA impairs homologous recombination repair, enhancing sensitivity to platinum-based agents, while hyperthermia further augments platinum uptake and DNA-damage lethality. BRCA1-mutated tumors appeared more beneficial in HIPEC indicated that platinum-responsive tumor microenvironment is a critical modifier of HIPEC effect, which is consistent with previous clinical trials. In OVHIPEC-1,^15^ adding cisplatin-based HIPEC at interval CRS after response to platinum-based neoadjuvant chemotherapy significantly improved recurrence-free survival and OS, with durable benefit confirmed on 10-year follow-up.^27^ In the recurrent setting, the phase III CHIPOR trial showed a significant OS advantage of HIPEC+ secondary CRS at the first platinum-sensitive relapse.^42^ Conversely, trials performing HIPEC at frontline surgery without confirmed platinum responsiveness found no significant survival advantage.^28^ BRCA2-mutated tumors already exhibit high platinum sensitivity, which attenuate the incremental advantage of hyperthermia. HIPEC also appeared more advantageous in BRCA wild-type patients, some of which have mutations in other homologous recombination related genes, and some of which are platinum resistant. In such cases, the hyperthermia and mechanical stimulation through fluid shear stress of HIPEC further improve cytotoxic efficacy compared to IP. Collectively, these exploratory findings indicate potential effect heterogeneity between HIPEC and IP, particularly in subgroups defined by tumor molecular features and perfusion timing, warranting further validation in prospective trials.

HIPEC was associated with higher incidences of AEs such as hypoalbuminemia, abdominal distension, and wound dehiscence. Hyperthermia promotes albumin loss through increasing peritoneal permeability.^45^ Heated perfusate (41-43 °C) can cause peritoneal inflammatory reactions and local tissue edema, contributing to abdominal distension. The high rate of severe wound dehiscence in the HIPEC group is partly attributed to the frequent treatment regimen and larger perfusate volume per session (4000 mL vs 500 mL). Notably, the AE profile observed in our study differs from previous reports, which often cited electrolyte imbalances, ileus, and urinary tract infections as dominant HIPEC complications.^8^^,^^28^ These findings highlighted the need for careful patient selection and optimized perioperative management when considering HIPEC. Patients with older age, high-grade serous histology, BRCA-wild/BRCA1-mutated, early initiation of treatment and suitable physical condition more likely to benefit from HIPEC than conventional IP. Meanwhile, attention to perioperative nutritional support, fluid balance, and wound care is essential to mitigate treatment-related toxicity and ensure the safety of HIPEC implementation in clinical practice.

As the first real-world study directly comparing HIPEC and conventional IP, this research provides a comprehensive evaluation of efficacy and safety, supported by multiple sensitivity analyses confirming the robustness of the findings. However, several limitations should be acknowledged. First, this study has limitations inherent in real-world studies, such as loss to follow-up and a large proportion of censored data, which affects data completeness and produces inaccuracies; Second, due to incomplete medical documentation, some clinical covariates such as AE grading, physical condition scores, ascites extent, and specific NACT information were unavailable or ambiguously recorded, which could not be adjusted for in the IPTW model; Third, as a single-center study, the generalizability of our findings may be limited by local perfusion devices and procedural details, including the use of lobaplatin and the concentration of perfusate. Therefore, our findings should be validated by future prospective multicenter studies with standardized regimens, ensuring uniform drug regimens, perfusate volume, and session number as well as extended safety follow-up.

## Supplementary Material

oyaf424_Supplementary_Data

## Data Availability

The raw data supporting the conclusions of this manuscript will be made available by the authors without undue reservation, to any qualified researcher. All data generated or analyzed during this study are included either in this article or available from the correspondence authors.
