# Supplementary material for: Real-world effectiveness and safety of hyperthermic intraperitoneal chemotherapy and intraperitoneal chemotherapy in ovarian cancer
Source: Oncologist. 2025 Dec 19;31(2):oyaf424. doi: 10.1093/oncolo/oyaf424 (PMC12811078; doi:10.1093/oncolo/oyaf424)
Supplement: oyaf424_Supplementary_Data [file oyaf424_supplementary_data.zip › Supplementary Figures.docx]

**
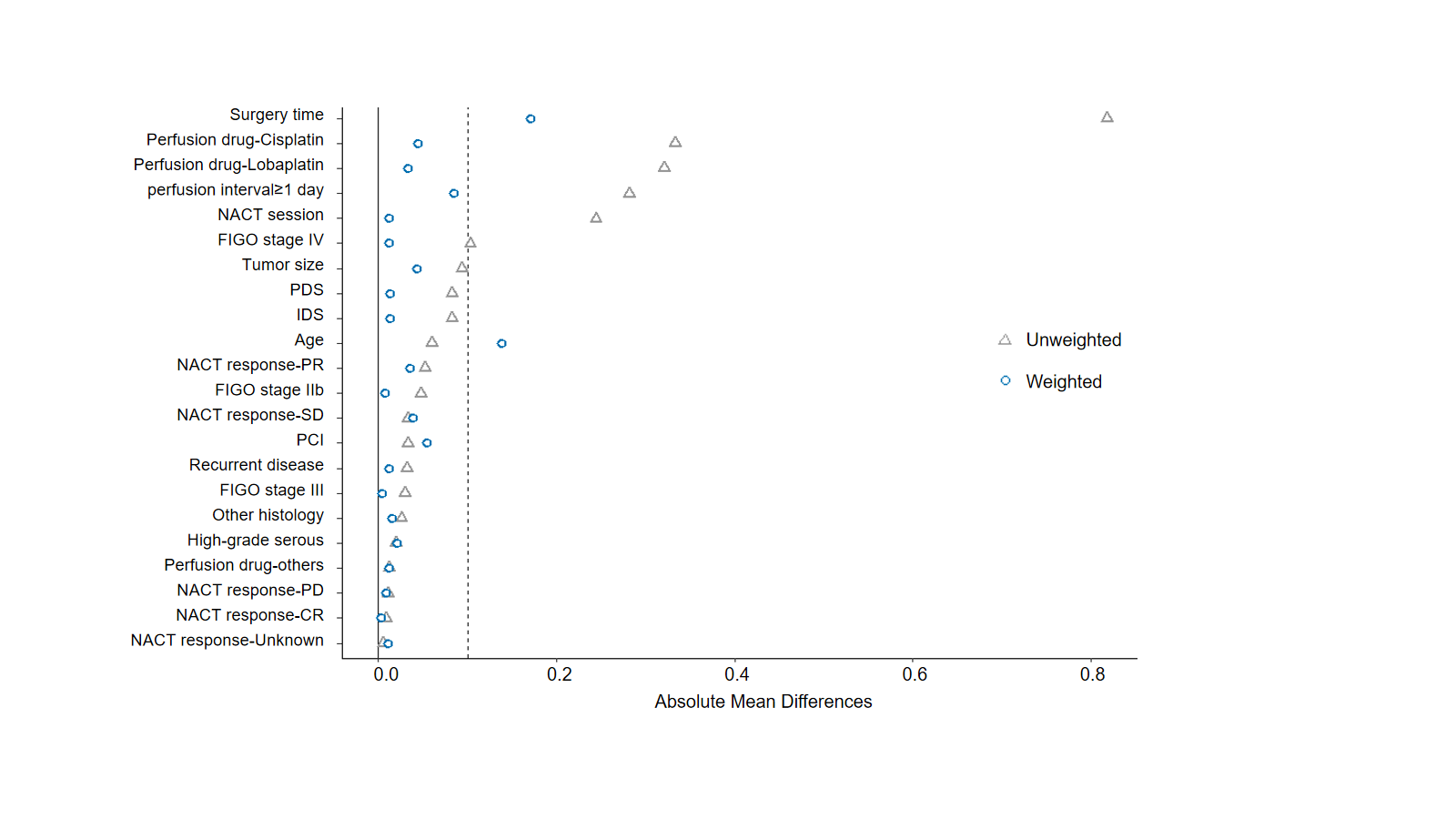
Figure S1. Standardized mean differences before and after weighting.**

Love plot showing standardized mean differences before and after weighting; most covariates achieved adequate balance (SMD < 0.1).

**Figure S2. Propensity score distribution.**

**
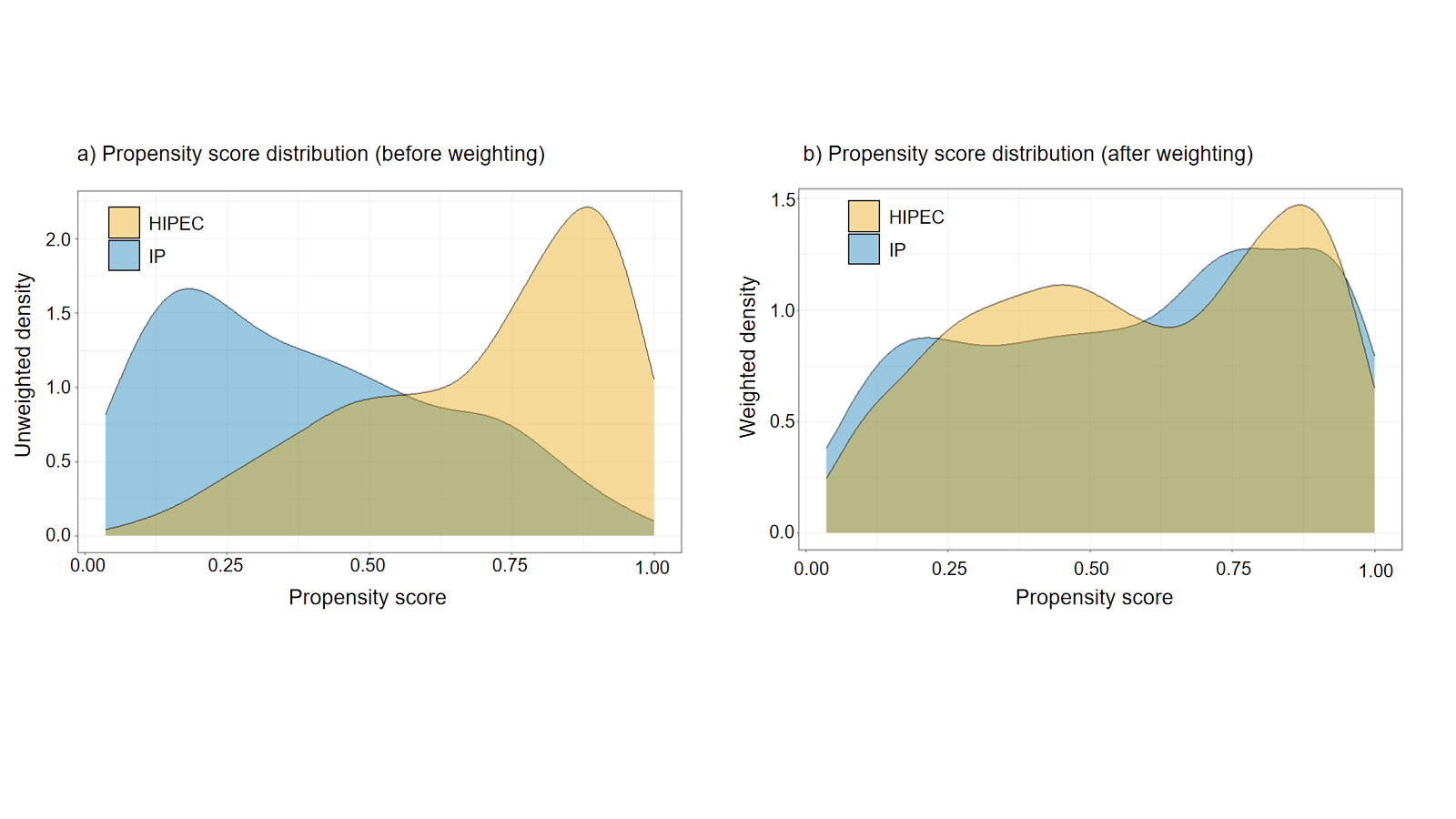
**

a) Propensity score distribution before weighting. b) Propensity score distribution after IPTW weighting showing improved overlap, demonstrating that weighting successfully balanced the distribution of observed covariates between treatment groups.

**Figure S3. Distribution of stabilized IPTW weights.**


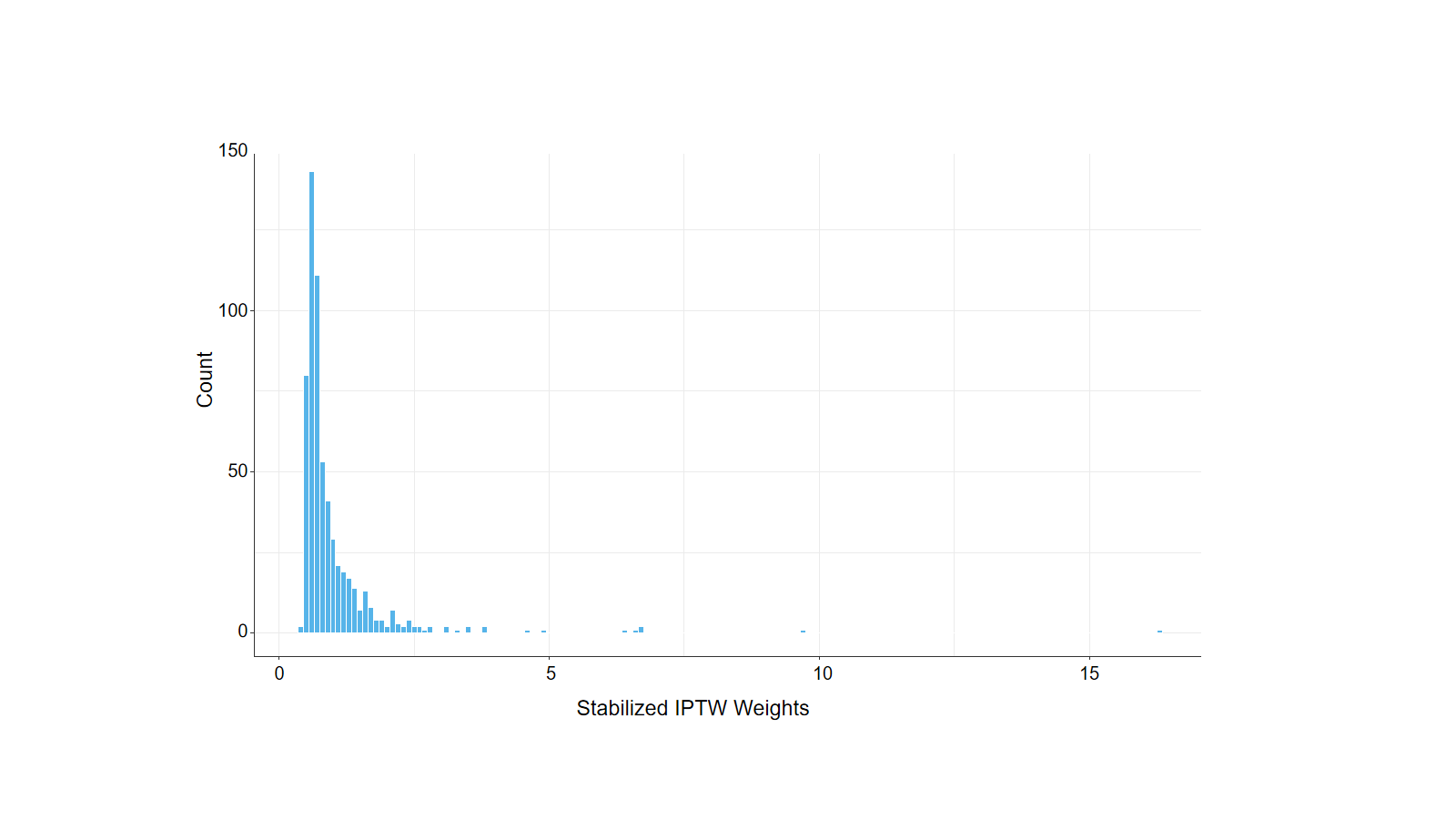


The histogram shows the distribution of stabilized IPTW weights. Most weights cluster between 0 and 3, with few extreme values (>10), indicating stable estimation.

**Figure S4. Additional subgroup analyses of progression-free survival.**


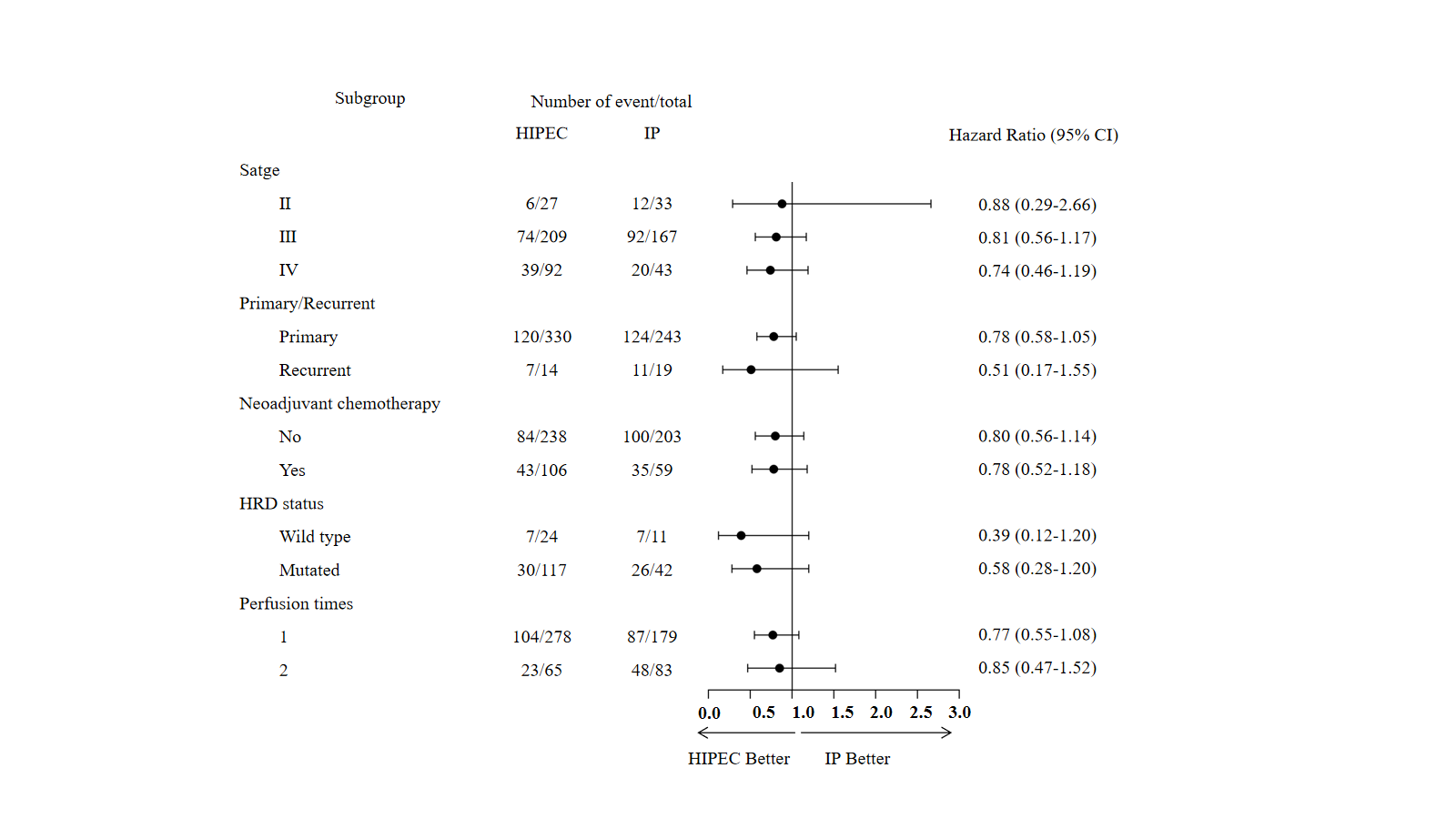


Subgroup analyses showing the associations between treatment strategy (HIPEC vs. IP) and progression-free survival across various clinicopathological subgroups. Circles represent hazard ratio point estimates comparing HIPEC with IP within each subgroup, and horizontal lines indicate the corresponding 95% confidence intervals.

**Figure S5. Distribution of propensity scores before and after matching.**


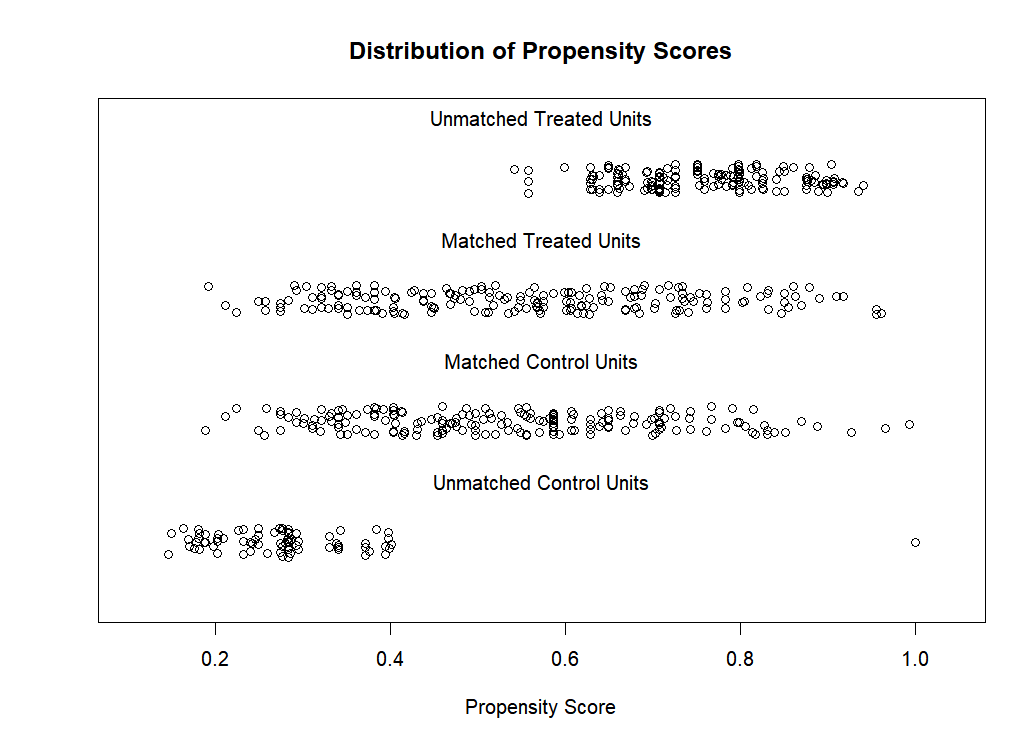


Distribution of propensity scores for HIPEC and IP group before and after matching. To reduce potential confounding and improve group comparability, propensity score matching was performed. A logistic regression model was used to estimate propensity scores with treatment group (HIPEC vs IP) as the outcome and relevant clinical covariates (stage, surgery time, and Treatment_Interval) as predictors. Nearest neighbor matching was applied at a 1:1 ratio using a caliper width of 0.2 to restrict poor matches. After matching, covariate balance between groups was assessed using standardized mean differences (SMD) with a threshold of <0.1 considered acceptable. Visual assessment of the matching quality was conducted using jitter plots of propensity scores. Matched two groups show balanced propensity score distribution, indicating adequate covariate matching, thereby reducing the interference of confounding factors on the estimation of treatment effects. The upper half represents the HIPEC group, and the lower half represents the IP group. The horizontal axis is the propensity score.


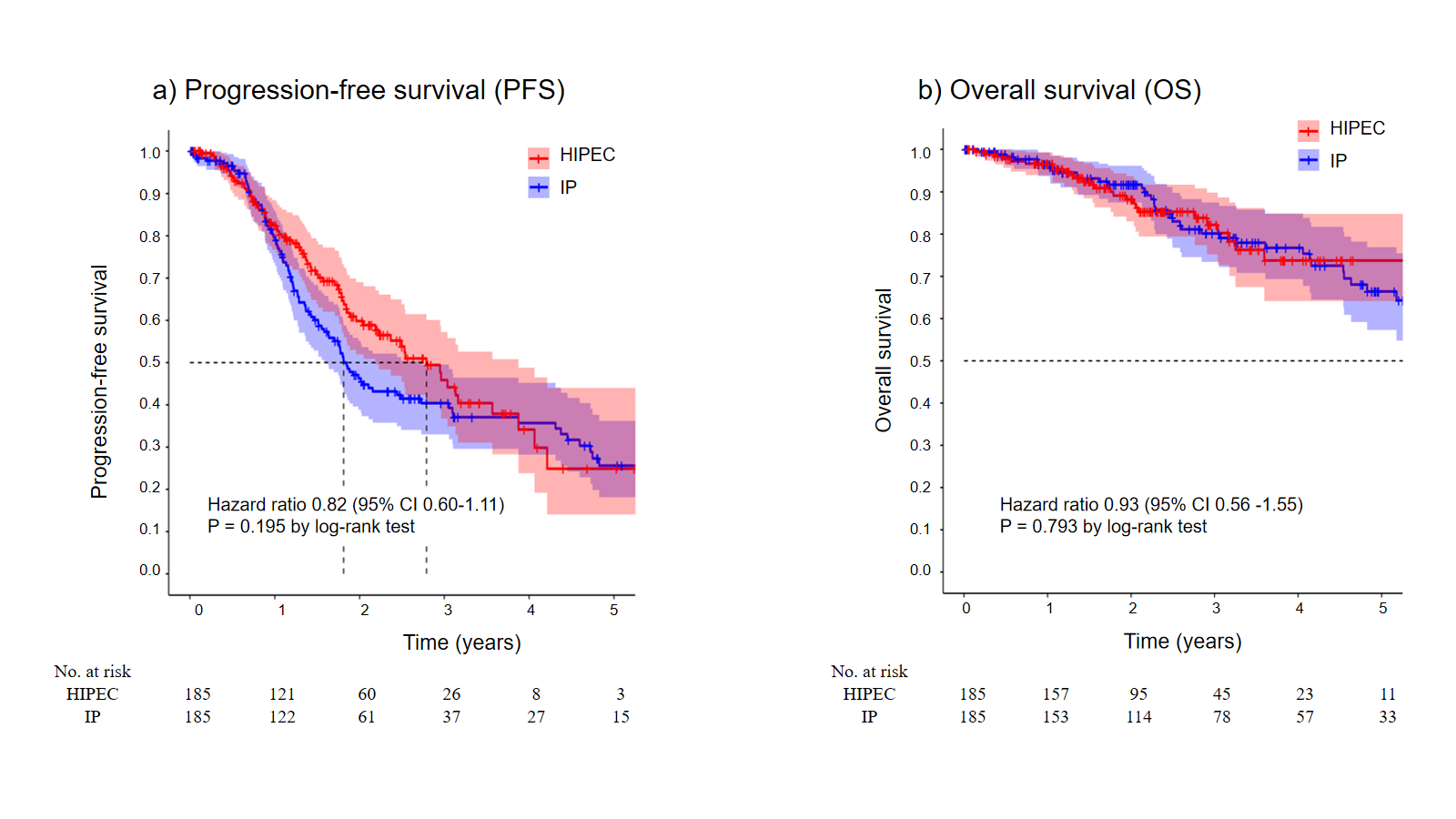
**Figure S6. Kaplan-Meier survival curves after propensity score matching.**

In progression-free survival (PFS), the HIPEC group showed a favorable trend compared to the IP group, but without statistical significance (p=0.195 by log-rank test); b) No significant difference was observed in overall survival (OS) between the two groups (p=0.793 by log-rank test). Shaded areas indicate 95% confidence intervals. The number-at-risk table below each curve represents patients still at risk at each time point.
