# Supplementary material for: Real-world effectiveness and safety of hyperthermic intraperitoneal chemotherapy and intraperitoneal chemotherapy in ovarian cancer
Source: Oncologist. 2025 Dec 19;31(2):oyaf424. doi: 10.1093/oncolo/oyaf424 (PMC12811078; doi:10.1093/oncolo/oyaf424)
Supplement: oyaf424_Supplementary_Data [file oyaf424_supplementary_data.zip › Supplementary tables.docx]

**Supplementary Content**

Catalogue

Table S1. Sugarbaker’s Peritoneal Carcinomatosis Index (PCI)2

Table S2. FIGO Staging Classification of Ovarian Cancer (1988)3

Table S3. The pathologist and the residual tumor (R) classification5

Table S4. Additional Demographic and Baseline Disease Characteristics and Treatment Information6

Table S5. Univariable and multivariable Cox proportional hazard regression models for PFS and OS10

Table S6. Additional Adverse Events12

This supplemental material has been provided by the authors to give readers additional information about their work.

| **Table S1. Sugarbaker’s Peritoneal Carcinomatosis Index (PCI)^[1]^** | |
| --- | --- |
| Score | Lesion size |
| 0 | No malignant deposits are visualized |
| 1 | Tumor nodules less than 0.5 are present |
| 2 | Tumor nodules between 0.5 and 5.0 cm are present; |
| 3 | Tumor nodules greater than 5.0 cm in any dimension, or in case there is conﬂuence of tumor. |

The summation of the Lesion Size score in each of the abdomino-pelvic regions is the PCI of the patient, with a maximal score of 39.

| **Table S2. FIGO Staging Classification of Ovarian Cancer (1988)^[2]^** | |
| --- | --- |
| Ⅰ | Growth limited to the ovaries |
| ⅠA | Tumor limited to one ovary; capsule intact, no tumor on ovarian surface; no malignant cells in ascites or peritoneal washings |
| ⅠB | Tumor limited to both ovaries; capsule intact, no tumor on ovarian surface; no malignant cells in ascites or peritoneal washings |
| ⅠC | Tumor limited to one or both ovaries; capsule ruptured, tumor on ovarian surface; malignant cells in ascites or peritoneal washings |
| Ⅱ | Tumor involves one or both ovaries with pelvic extension |
| ⅡA | Extension and/or implants on uterus and/or tube(s) |
| ⅡB | Extension to other pelvic tissues |
| ⅡC | Pelvic extension with any of the following: capsule ruptured, tumor on ovarian surface, malignant cells in ascites or peritoneal washings |
| Ⅲ | Tumor involves one or both ovaries with peritoneal metastasis outside the pelvis and/or retroperitoneal or inguinal lymph node metastasis |
| ⅢA | Microscopic peritoneal metastasis beyond pelvis |
| ⅢB | Microscopic peritoneal metastasis beyond pelvis 2cm or less in greatest dimension |
| ⅢC | Peritoneal metastasis beyond pelvis more than 2cm in greatest dimension and/or positive retroperitoneal or inguinal lymph nodes |
| Ⅳ | Growth involving one or both ovaries with distant metastases. If pleural effusion is present, there must be positive cytology to allot a case to Stage IV. Parenchymal liver metastasis equals Stage IV |
| ⅣA | Pleural effusion with positive cytology |
| ⅣB | Parenchymal metastases and metastases to extra-abdominal organs(including inguinal lymph nodes and lymph nodes outside of the abdominal cavity) |
| Only patients with FIGO stage ≥ IIB were included in the present study.  Abbreviation: FIGO, International Federation of Gynecology and Obstetrics. | |

**Table S3. The pathologist and the residual tumor (R) classification^[3]^**

| R0 | No macroscopic residual disease |
| --- | --- |
| R1 | Single residual tumor lesion with maximum diameter of ≤ 1 cm |
| R2 | Single residual tumor lesion with maximum diameter of >1 cm |

The optimal resection was defined as the combination of R0 and R1.

| **Table S4. Additional demographic and baseline disease characteristics and treatment information ^a^** | | | | | | |
| --- | --- | --- | --- | --- | --- | --- |
|  | Cohort before Inverse Probability of Treatment Weighting | | | Cohort after Inverse Probability of Treatment Weighting | | |
| **Variable** | HIPEC  (N=344) | IP  （N=262） | *p* value ^b^ | HIPEC  (N=335) | IP  （N=269） | *p* value ^c^ |
| Median Gravidity (IQR), times | 2 (1-3) | 3 (2-4) | 0.001 | 2 (1-3) | 3 (2-4) | 0.001 |
| Median Parity (IQR), times | 2 (1-2) | 2 (1-2) | 0.180 | 1 (1-2) | 2 (1-2) | 0.098 |
| Pausimenia |  |  | 1.000 |  |  | 0.680 |
| Yes | 247 (71.8) | 188 (71.8) |  | 239 (71.3) | 197 (73.4) |  |
| No | 97 (28.2) | 74 (28.2) |  | 96 (28.7) | 71 (26.6) |  |
| Median weight (IQR), kg | 54.0 (49.7-60.0) | 54.0 (47.5-58.9) | 0.087 | 54.5 (50.0-60.0) | 54.5 (48.9-60.0) | 0.489 |
| Median height (IQR), cm | 158.0 (154.9-162.0) | 159.0 (155.0-162.0) | 0.256 | 159.0 (155.0-162.0) | 159.0 (153.6-163.0) | 0.869 |
| Median preoperative CA125 value (IQR), U/ml | 231.0 (51.1-731.5) | 312.1 (58.4-1095.0) | 0.201 | 208.4 (58.6-692.9) | 203.6 (47.6-1035.6) | 0.784 |
| Median preoperative HE4 value (IQR), U/ml | 195.4 (98.0-479.6) | 238.8 (103.7-538.1) | 0.253 | 211.9 (92.9-474.7) | 206.9 (99.9-522.6) | 0.772 |
| FIGO stage IV ^d^ , no. (%) |  |  | 0.164 |  |  | 0.001 |
| IVa | 15 (21.4) | 2 (7.1) |  | 13 (22.1) | 1 (2.4) |  |
| IVb | 55 (78.6) | 26 (92.9) |  | 45 (77.9) | 51 (97.6) |  |
| Platinum sensitivity ^e^, no. (%) |  |  | 0.737 |  |  | 0.331 |
| Platinum-sensitive | 10 (66.7) | 17 (77.3) |  | 20 (71.6) | 17 (85.9) |  |
| Platinum-resistant | 5 (33.3) | 5 (22.7) |  | 8 (28.4) | 3 (14.1) |  |
| BRCA status, no. (%) |  |  | <0.001 |  |  | 0.032 |
| Wild type | 143 (41.6) | 39 (14.9) |  | 131 (39.1) | 57 (21.4) |  |
| BRCA1 | 45 (13.1) | 25 (9.5) |  | 40 (11.8) | 29 (10.8) |  |
| BRCA2 | 13 (3.8) | 10 (3.8) |  | 13 (3.7) | 15.4 (5.7) |  |
| Unknown | 143 (41.6) | 188 (71.8) |  | 152 (45.3) | 167 (62.1) |  |
| HRD status, no. (%) |  |  | <0.001 |  |  | 0.332 |
| Wild type | 24 (7.0) | 11 (4.2) |  | 28 (8.4) | 25 (9.3) |  |
| Mutated | 117 (34.0) | 42 (16.0) |  | 106 (31.6) | 55 (20.6) |  |
| Unknown | 203 (59.0) | 209 (79.8) |  | 201 (60.0) | 189 (70.2) |  |
| Neoadjuvant chemotherapy, no. (%) |  |  | 0.029 |  |  | 0.835 |
| Yes | 106 (30.8) | 59 (22.5) |  | 87 (25.8) | 66 (24.5) |  |
| No | 238 (69.2) | 203 (77.5) |  | 249 (74.2) | 203 (75.5) |  |
| Neoadjuvant chemotherapy response, no. (%) |  |  | 0.053 |  |  | 0.500 |
| CR | 1 (0.3) | 3 (1.1) |  | 1 (0.3) | 2 (0.5) |  |
| PR | 47 (13.7) | 22 (8.4) |  | 38 (11.3) | 21 (7.8) |  |
| SD | 22 (6.4) | 8 (3.1) |  | 17 (5.0) | 24 (8.9) |  |
| PD | 9 (2.6) | 4 (1.5) |  | 6 (1.7) | 2 (0.9) |  |
| Unknown | 27 (7.8) | 22 (8.4) |  | 25 (7.4) | 17 (6.4) |  |
| Median postoperative intravenous chemotherapy (IQR), courses | 6 (4-6) | 6 (4-6) | 0.843 | 6(4-6) | 6(4-6) | 0.778 |
| Median time from CRS to first intravenous chemotherapy, days | 8 (7-10) | 8 (6-11) | 0.103 | 8 (7-11) | 8 (7-11) | 0.348 |
| Maintenance treatment, no. (%) |  |  | <0.001 |  |  | 0.009 |
| Bevacizumab | 4 (1.5) | 25 (7.3) |  | 19 (7.2) | 20 (5.8) |  |
| PARP inhibitor | 16 (6.1) | 75 (21.8) |  | 15 (5.6) | 69 (20.4) |  |
| Bevacizumab+PARP inhibitor | 1 (0.4) | 22 (6.4) |  | 1 (0.3) | 23 (6.8) |  |
| Unknown/ None | 241 (92.0) | 222 (64.5) |  | 234 (86.9) | 225 (66.9) |  |
| Progression evidence, no. (%) |  |  | 0.909 |  |  | 0.142 |
| Radiologic findings | 91 (71.7) | 92 (70.2) |  | 102 (75.0) | 88 (61.7) |  |
| Biochemical assessment | 36 (28.3) | 39 (29.8) |  | 34 (25.0) | 55 (38.3) |  |

^a^ Due to rounding error, in the weighted cohort counts may not sum to expected totals, and percentages may not be equal to ratios of counts.

^b^ P values calculated from chi-square test or Wilcoxon rank-sum test.

^c^ P values calculated from inverse probability of treatment-weighted logistic regression models.

^d^ Stage IV disease was subclassified as IVA (pleural effusion with positive cytology) and IVB (parenchymal or extra-abdominal metastases). There was unknown in 37 cases due to incomplete documentation.

^e^ Recurrent ovarian cancer was classified into platinum-sensitive (platinum-free interval ≥ 6 months) and platinum-resistant (platinum-free interval < 6 months).

Abbreviation: HIPEC, hyperthermic intraperitoneal chemotherapy; IP, intraperitoneal chemotherapy; IQR, interquartile range; CR, complete response; PR, partial response; SD, stable disease; PD, progressive disease.

**Table S5. Univariable and multivariable Cox proportional hazard regression models for PFS and OS**

| Variables |  | Progression-Free Survival (PFS) | | | | Overall Survival (OS) | | | |
| --- | --- | --- | --- | --- | --- | --- | --- | --- | --- |
|  |  | Univariable model |  | Multivariable model |  | Univariable model |  | Multivariable model |  |
|  |  | HR (95% CI) | *p* value | HR (95% CI) | *p* value | HR (95% CI) | *p* value | HR (95% CI) | *p* value |
| Group |  |  |  |  |  |  |  |  |  |
|  | IP | 1(ref) |  | 1(ref) |  | 1(ref) |  | 1(ref) |  |
|  | HIPEC | 0.911(0.71-1.169) | 0.466 | 0.756(0.571-1.001) | 0.05 | 1.023(0.688-1.519) | 0.912 | 0.952(0.625-1.450) | 0.819 |
| Age, years |  | 1.017(1.004-1.029) | 0.007 | 1.017(1.003-1.031) | 0.014 | 1.04(1.02-1.061) | 0.00 | 1.034(1.012-1.057) | 0.002 |
| FIGO Stage |  |  |  |  |  |  |  |  |  |
|  | Ⅱ | 1(ref) |  | 1(ref) |  | 1(ref) |  | 1(ref) |  |
|  | Ⅲ | 1.898(1.165-3.091) | 0.01 | 1.793(1.096-2.932) | 0.02 | 2.552(1.021-6.381) | 0.045 | 1.834(0.705-4.769) | 0.214 |
|  | Ⅳ | 2.133(1.255-3.626) | 0.005 | 1.937(1.082-3.469) | 0.026 | 4.942(1.912-12.778) | 0.001 | 3.306(1.153-0.477) | 0.026 |
| Histology |  |  |  |  |  |  |  |  |  |
|  | High-grade serous | 1(ref) |  |  |  | 1(ref) |  |  |  |
|  | Others | 0.824(0.576-1.178) | 0.288 |  |  | 1.054(0.639-1.738) | 0.838 |  |  |
| Neoadjuvant chemotherapy |  |  |  |  |  |  |  |  |  |
|  | No | 1(ref) |  | 1(ref) |  | 1(ref) |  | 1(ref) |  |
|  | Yes | 1.439(1.101-1.880) | 0.008 | 1.126(0.802-1.583) | 0.493 | 1.75（1.172-2.612） | 0.006 | 1.227(0.707-2.218) | 0.467 |
| PCI |  | 1.004(0.979-1.030) | 0.747 |  |  | 1.043(1.004-1.083) | 0.031 |  |  |
| Primary/Recurrent |  |  |  |  |  |  |  |  |  |
|  | Primary | 1(ref) |  |  |  | 1(ref) |  |  |  |
|  | Recurrent | 1.845(1.142-2.982) | 0.012 |  |  | 1.739(0.878-3.447) | 0.113 |  |  |
| Interval between surgery and start of HIPEC/IP, days |  |  |  |  |  |  |  |  |  |
|  | ≤1 | 1(ref) |  | 1(ref) |  | 1(ref) |  |  |  |
|  | >1 | 0.765(0.598-0.979) | 0.033 | 0.653(0.496-0.859) | 0.002 | 0.714(0.487-1.046) | 0.084 |  |  |
| Surgery duration, minutes |  | 1.001(1.000-1.003) | 0.155 |  |  | 1.002(1.000-1.003) | 0.073 |  |  |

Univariate analysis was performed to screen variables and then significant variables (P < 0.05) were included in the multivariate analysis. Baseline characteristics, including histology, residual disease after CRS, primary/ recurrent or surgery duration were not significant for survival outcomes in the multivariable Cox proportional hazards regression model. After adjusting PFS by age, stage, presence or absence of neoadjuvant chemotherapy and interval time between surgery and initiation of treatment, HIPEC yielded a HR of 0.76 (95% CI 0.57-1.00; p=0.05) compared to IP. Abbreviation: HR, hazard ratio; CI, confidence interval; HIPEC, hyperthermic intraperitoneal chemotherapy; IP, intraperitoneal chemotherapy.

**Table S6. Additional adverse events**

| Adverse events | Any grade | | | | Grade 3 or 4 | | |
| --- | --- | --- | --- | --- | --- | --- | --- |
|  | No. (%) | | *p* value | No. (%) | | | *p* value |
|  | HIPEC  (N=335) | IP  （N=269） |  | HIPEC  (N=335) | | IP  （N=269） |  |
| Diarrhea | 3 (1) | 5 (1.9) | 0.317 | 1 (0.2) | | 1 (0.5) | 0.622 |
| chest distress | 5 (1.5) | 3 (1.3) | 0.901 | 0 | | 0 |  |
| Palpitations | 5 (1.4) | 3 (1.1) | 0.852 | 0 | | 0 |  |
| Chills | 3 (0.9) | 2 (0.8) | 0.892 | 0 | | 0 |  |
| Rash | 0 | 4 (1.6) | 0.056 | 0 | | 1 (0.2) | 0.268 |
| Syncope | 2 (0.6) | 0 | 0.369 | 2 (0.6) | | 0 | 0.369 |
| Delirium | 2 (0.5) | 1 (0.2) | 0.383 | 0 | | 0 |  |
| Wheezing | 3 (0.8) | 0 | 0.081 | 0 | | 0 |  |
| Depressed level of consciousness | 1 (0.4) | 0 | 0.371 | 1 (0.4) | | 0 | 0.371 |
| acute kidney injury | 1 (0.4) | 0 | 0.371 | 0 | | 0 |  |
| bleeding | 0 | 1 (0.3) | 0.268 | 0 | | 0 |  |
| Chest pain | 1 (0.2) | 0 | 0.373 | 0 | | 0 |  |
| Dizziness | 1 (0.3) | 0 | 0.373 | 0 | | 0 |  |
| Malaise | 1 (0.2) | 0 | 0.373 | 0 | | 0 |  |
| Somnolence | 1 (0.2) | 0 | 0.373 | 0 | | 0 |  |
| uroclepsia | 1 (0.2) | 0 | 0.373 | 0 | | 0 |  |
| Ileus | 1 (0.2) | 0 | 0.373 | 0 | | 0 |  |

Group differences of weighted event rates estimated using survey-adjusted means and performed weighted chi-square tests.

Abbreviation: HIPEC, hyperthermic intraperitoneal chemotherapy; IP, intraperitoneal chemotherapy.

1 Portilla AG, Shigeki K, Dario B, et al. The intraoperative staging systems in the management of peritoneal surface malignancy. J Surg Oncol 2008;98:228-231.

2 Pecorelli S, Benedet JL, Creasman WT, et al. Figo staging of gynecologic cancer. 1994-1997 figo committee on gynecologic oncology. International federation of gynecology and obstetrics. Int J Gynaecol Obstet 1999;64:5-10.

3 Shih KK and Chi DS. Maximal cytoreductive effort in epithelial ovarian cancer surgery. J Gynecol Oncol 2010;21:75-80.
